# Composition and Structure of *Pinus koraiensis* Mixed Forest Respond to Spatial Climatic Changes

**DOI:** 10.1371/journal.pone.0097192

**Published:** 2014-05-08

**Authors:** Jingli Zhang, Yong Zhou, Guangsheng Zhou, Chunwang Xiao

**Affiliations:** 1 State Key Laboratory of Vegetation and Environmental Change, Institute of Botany, the Chinese Academy of Sciences, Beijing, China; 2 University of Chinese Academy of Sciences, Beijing, China; 3 Chinese Academy of Meteorological Sciences, Beijing, China; Chinese Academy of Sciences, China

## Abstract

**Background:**

Although some studies have indicated that climate changes can affect *Pinus koraiensis* mixed forest, the responses of composition and structure of *Pinus koraiensis* mixed forests to climatic changes are unknown and the key climatic factors controlling the composition and structure of *Pinus koraiensis* mixed forest are uncertain.

**Methodology/principal findings:**

Field survey was conducted in the natural *Pinus koraiensis* mixed forests along a latitudinal gradient and an elevational gradient in Northeast China. In order to build the mathematical models for simulating the relationships of compositional and structural attributes of the *Pinus koraiensis* mixed forest with climatic and non-climatic factors, stepwise linear regression analyses were performed, incorporating 14 dependent variables and the linear and quadratic components of 9 factors. All the selected new models were computed under the +2°C and +10% precipitation and +4°C and +10% precipitation scenarios. The Max Temperature of Warmest Month, Mean Temperature of Warmest Quarter and Precipitation of Wettest Month were observed to be key climatic factors controlling the stand densities and total basal areas of *Pinus koraiensis* mixed forest. Increased summer temperatures and precipitations strongly enhanced the stand densities and total basal areas of broadleaf trees but had little effect on *Pinus koraiensis* under the +2°C and +10% precipitation scenario and +4°C and +10% precipitation scenario.

**Conclusions/significance:**

These results show that the Max Temperature of Warmest Month, Mean Temperature of Warmest Quarter and Precipitation of Wettest Month are key climatic factors which shape the composition and structure of *Pinus koraiensis* mixed forest. Although the *Pinus koraiensis* would persist, the current forests dominated by *Pinus koraiensis* in the region would all shift and become broadleaf-dominated forests due to the dramatic increase of broadleaf trees under the future global warming and increased precipitation.

## Introduction

*Pinus koraiensis* is a pine species endemic to the region including East Russia, Korea, Japan and Northeast China [1], [2], which provides edible seeds and very useful timber [2], and usually forms mixed forest with various broadleaf trees [3]. The *Pinus koraiensis* - broadleaf mixed forest is one of the major vegetation types and the typical conifer - broadleaf mixed forest and plays important role in carbon cycling in Northeast China [3], [4].

A field study of the North East China Transect (NECT) has observed the shrinkage of *Pinus koraiensis* patches and the expansion of some broadleaf tree species from 1986 to 1994, and indicated that the reason for the shrinkage of *Pinus koraiensis* patches and the expansion of some broadleaf tree species might be the effects of climate change [5]. Some simulation studies also showed that climate changes could affect *Pinus koraiensis* or its mixed forest [6], [7]. For example, by using an ecological information system (GREEN), Xu and Yan (2001) predicted that the potential distribution area of *Pinus koraiensis* would shift Northward under the HadCM2 climatic change scenario and under the average of another five climatic change scenarios (GISS, NCAR, OSU, UKMO and MPI) [6]. By linking a forest gap model (LINKAGES) with a landscape model (LANDIS), He et al. (2005) predicted that the broadleaf trees would overtake *Pinus koraiensis* to become broadleaf forests in Changbai Natural Reserve of Northeast China under the CGCM2 climate change scenario [7]. However, the key climatic factors controlling the composition and structure of *Pinus koraiensis* mixed forest are uncertain.

Linear or quadratic regression models can be built to simulate the relationships of compositional and structural attributes of plant communities with climatic and non-climatic factors, and such studies have been conducted in grasses, tropical trees, *Pinus contorta* and *Pinus sylvestris* [8], [9], [10], [11], [12]. To better understand the compositional and structural changes of plant communities, it is necessary to recognize the effects of natural succession with age, which strongly affect the compositional and structural changes of plant communities even without evidence of climatic change [13], [14], [15]. Usually, the mean diameter of *Pinus koraiensis* reflects the stand age of a *Pinus koraiensis* mixed forest, because the mean diameter of *Pinus koraiensis* in a stand naturally increases over time [16].

According to the recent CMIP5 (phase five of the Coupled Model Inter-comparison Project) climate change scenarios, the global temperature averaged in 2081–2100 is projected to likely exceed 2°C (RCP6.0 and RCP8.5 emission scenarios) above 1850–1900; although unlikely to exceed 4°C in all other RCP emission scenarios than RCP8.5 [17]. The recent CMIP5 scenarios also predicted that not only the surface air temperature but also the precipitation in 2071–2100 would be higher than those in 1986–2005 in Northeast China [18].

To examine the responses of composition of and structure of *Pinus koraiensis* mixed forests to spatial climatic changes, a field survey was conducted in the natural *Pinus koraiensis* mixed forests along a latitudinal gradient and an elevational gradient in Northeast China. The mathematical models were built for simulating the relationships of compositional and structural attributes of the *Pinus koraiensis* mixed forest with climatic and non-climatic factors, and two scenarios of the +2°C and +10% precipitation and +4°C and +10% precipitation were used to predict the possible changes of composition and structure in *Pinus koraiensis* mixed forests under the future global climatic change. The objectives of this study are to: (1) build mathematical models to quantify the responses of the composition and structure of *Pinus koraiensis* mixed forests to the climatic factors; (2) discover the key climatic factors which control the composition and structure of *Pinus koraiensis* mixed forest. Additionally, we hypothesized that the composition and structure of *Pinus koraiensis* mixed forests would significantly be affected and *Pinus koraiensis* mixed forests would be overtaken by broadleaf-dominated forests in the region under the future global warming and increased precipitation.

## Materials and Methods

### Ethics Statement

All necessary permits were obtained for the described field studies. This study was approved by State Key Laboratory of Vegetation and Environmental Change, Institute of Botany, the Chinese Academy of Sciences; Shengshan National Nature Reserve; Liangshui National Nature Reserve; Dongsheng Forestry Bureau; Changbai National Nature Reserve and Kuandian National Nature Reserve.

### Study Area

**Figure 1:**
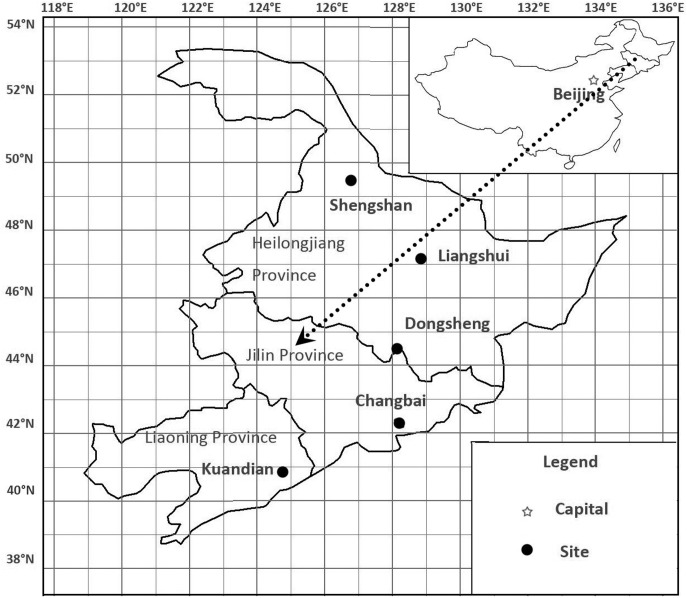
Location of study sites in Northeast China.

This study was conducted in the Augusts of 2010 and 2012 in Northeast China, including ten sites: Kuandian, Dongsheng, Liangshui, Shengshan and Changbai from A to F (Fig. 1). The former four sites formed a latitudinal gradient while the latter six sites formed an elevational gradient. Each site consists of three plots (Table 1). All the 30 plots are natural *Pinus koraiensis* mixed forests without recent disturbance. All the broadleaved trees are deciduous and most of the conifers are evergreen (except for *Larix*) in the study region. From June to August is the warmest quarter (summer) and January is the coldest month of the year in the study region. Rainy season begins at May or June and ends at September, and the summer (from June to August) precipitation occupies 60% of annual precipitation [19]. The 1-km-resolution bioclimate data of each plot were downloaded from WorldClim [20] (Table 2).

**Table 1:** Location and elevation of *Pinus koraiensis* mixed forests in different sites and plots.

Site	Plots	Plot Latitude (°N)	Plot Longitude (°E)	Plot Elevation (m)
Kuandian	Three 30 m * 20 m plots	40.9123	124.7883	930
Dongsheng	Three 30 m * 20 m plots	44.4113	128.1233	804
Liangshui	Three 30 m * 20 m plots	47.1662	128.8843	445
Shengshan	Three 20 m * 20 m plots	49.4780	126.7782	504
Changbai A	Three 30 m * 20 m plots	42.3993	128.0946	755
Changbai B	Three 30 m * 20 m plots	42.4001	128.0952	759
Changbai C	Three 30 m * 20 m plots	42.3742	128.0852	851
Changbai D	Three 30 m * 20 m plots	42.2655	128.1476	960
Changbai E	Three 30 m * 20 m plots	42.2270	128.1743	1058
Changbai F	Three 30 m * 20 m plots	42.1914	128.3117	1076

**Table 2:** The 23 Variables and their abbreviations used in the study.

Variable	Abbreviation
Max Temperature of Warmest Month (°C)	TempWarmestMonth
Min Temperature of Coldest Month (°C)	TempColdestMonth
Mean Temperature of Warmest Quarter (°C)	TempWarmestQuarter
Mean Temperature of Coldest Quarter (°C)	TempColdestQuarter
Precipitation of Wettest Month (mm)	PrecipWettestMonth
Precipitation of Driest Month (mm)	PrecipDriestMonth
Precipitation of Wettest Quarter (mm)	PrecipWettestQuarter
Precipitation of Driest Quarter (mm)	PrecipDriestQuarter
Mean DBH of *Pinus koraiensis* (cm)	MeanDBH_Pinus_
Total Basal Area of *Pinus koraiensis* (m^2^ ha^−1^)	BasalArea_Pinus_
Stand Density of *Pinus koraiensis* (No. ha^−1^)	StandDensity_Pinus_
Total Basal Area of *Acer* and *Fraxinus* (m^2^ ha^−1^)	BasalArea_Acer-Fraxinus_
Stand Density of *Acer* and *Fraxinus* (No. ha^−1^)	StandDensity_Acer-Fraxinus_
Total Basal Area of *Quercus* (m^2^ ha^−1^)	BasalArea_Quercus_
Stand Density of *Quercus* (No. ha^−1^)	StandDensity_Quercus_
Total Basal Area of *Abies* and *Picea* (m^2^ ha^−1^)	BasalArea_Abies-Picea_
Stand Density of *Abies* and *Picea* (No. ha^−1^)	StandDensity_Abies-Picea_
Total Basal Area of *Larix* (m^2^ ha^−1^)	BasalArea_Larix_
Stand Density of *Larix* (No. ha^−1^)	StandDensity_Larix_
Total Basal Area of *Ulmus* (m^2^ ha^−1^)	BasalArea_Ulmus_
Stand Density of *Ulmus* (No. ha^−1^)	StandDensity_Ulmus_
Total Basal Area of *Betula* (m^2^ ha^−1^)	BasalArea_Betula_
Stand Density of *Betula* (No. ha^−1^)	StandDensity_Betula_

### Field Survey and Data Analyses

In each plot, every living tree higher than 1.4 m was identified to species and then measured for its circumference with a tape measure at its breast height (1.3 m). The measured circumference of each tree was converted into its diameter at breast height (DBH) and calculated into its basal area using the circle formulas.

Fifteen attributes of *Pinus koraiensis* mixed forest were extracted from each plot. The fifteen attributes were: Mean DBH of *Pinus koraiensis* (MeanDBH_Pinus_), Total Basal Area of *Pinus koraiensis* (BasalArea_Pinus_), Stand Density of *Pinus koraiensis* (StandDensity_Pinus_), Total Basal Area of *Acer* and *Fraxinus* (BasalArea_Acer-Fraxinus_), Stand Density of *Acer* and *Fraxinus* (StandDensity_Acer-Fraxinus_), Total Basal Area of *Quercus* (BasalArea_Quercus_), Stand Density of *Quercus* (StandDensity_Quercus_), Total Basal Area of *Abies* and *Picea* (BasalArea_Abies-Picea_), Stand Density of *Abies* and *Picea* (StandDensity_Abies-Picea_), Total Basal Area of *Larix* (BasalArea_Larix_), Stand Density of *Larix* (StandDensity_Larix_), Total Basal Area of *Ulmus* (BasalArea_Ulmus_), Stand Density of *Ulmus* (StandDensity_Ulmus_), Total Basal Area of *Betula* (BasalArea_Betula_) and Stand Density of *Betula* (StandDensity_Betula_) (Table 2).

One-way ANOVA and correlation and regression analyses were performed using SPSS 13.0. Standard errors within sites were detected by one-way ANOVA with descriptive options. Significant differences (p<0.05) between sites were detected using One-way ANOVA with post-Duncan's test.

Stepwise linear regression analyses were performed in order to generate new mathematical models for the *Pinus koraiensis* mixed forest. The independent factors used in the regressions were linear and quadratic components of Max Temperature of Warmest Month (TempWarmestMonth), Min Temperature of Coldest Month (TempColdestMonth), Mean Temperature of Warmest Quarter (TempWarmestQuarter), Mean Temperature of Coldest Quarter (TempColdestQuarter), Precipitation of Wettest Month (PrecipWettestMonth), Precipitation of Driest Month (PrecipDriestMonth), Precipitation of Wettest Quarter (PrecipWettestQuarter), Precipitation of Driest Quarter (PrecipDriestQuarter) and MeanDBH_Pinus_. The 14 dependent variables were BasalArea_Pinus_, StandDensity_Pinus_, BasalArea_Acer-Fraxinus_, StandDensity_Acer-Fraxinus_, BasalArea_Quercus_, StandDensity_Quercus_, BasalArea_Abies-Picea_, StandDensity_Abies-Picea_, BasalArea_Larix_, StandDensity_Larix_, BasalArea_Ulmus_, StandDensity_Ulmus_, BasalArea_Betula_ and StandDensity_Betula_ (Table 2). Regression models with non-significant (p>0.05) term(s) were discarded. Among candidate models with all terms significant (p<0.05), the model with the lowest Small Sample Unbiased Akaike Information Criterion (AICc) value was selected [21].

Two-tailed partial correlation coefficients between each dependent variable and the independent variables excluded from its regression model were tested, controlling for the independent variable(s) included in its regression model.

### Running the Model

The selected models were computed using the currently observed MeanDBH_Pinus_ values under current climate, under the +2°C and +10% precipitation scenario and under the +4°C and +10% precipitation scenario. In order to examine the concordances between the simulated and observed values under current climate, scatter plots with simple error bars and regressions were generated using Sigmaplot 10.0. To represent the variations of stand densities and basal areas of *Pinus koraiensis*, other conifers and broadleaves under current climate under the +2°C and +10% precipitation scenario and under the +4°C and +10% precipitation scenario, vertical bar charts with error bars were generated using Sigmaplot 10.0.

## Results

### Structure and Composition of *Pinus Koraiensis* Forest

Significant effects with latitude and/or elevation were observed in most of the compositional and structural attributes of *Pinus koraiensis* mixed forest (P<0.05, Table 3). The StandDensity_Pinus_ significantly increased while BasalArea_Ulmus_, StandDensity_Ulmus_, BasalArea_Betula_, StandDensity_Betula_, BasalArea_Acer-Fraxinus_ and StandDensity_Acer-Fraxinus_ significantly declined with increasing latitude (from the southernmost site Kuandian to the northernmost site Shengshan) (P<0.05, Table 3). BasalArea_Larix_, StandDensity_Larix_, BasalArea_Abies-Picea_ and StandDensity_Abies-Picea_ were significantly enhanced with increasing elevation (from Changbai A to Changbai F) (P<0.05, Table 3).

**Table 3:** Composition and structure of *Pinus koraiensis* mixed forests in different sites.

Site	MeanDB H_Pinus_ (cm)	BasalAre a_Pinus_ (m^2^ ha^−1^)	StandDensity_Pinus_ (No. ha^−1^)	BasalArea_Ulmus_ (m^2^ ha^−1^)	StandDensity_Ulmus_ (No. ha^−1^)	BasalArea_Betula_ (m^2^ ha^−1^)	StandDensity_Betula_ (No. ha^−1^)	BasalArea_Acer-Fraxinus_ (m^2^ ha^−1^)	StandDensity_Acer-Fraxinus_ (No. ha^−1^)	BasalArea_Larix_ (m^2^ ha^−1^)	StandDensity_Larix_ (No. ha^−1^)	BasalArea_Abies-Picea_ (m^2^ ha^−1^)	StandDensity_Abies-Picea_ (No. ha^−1^)	BasalArea_Quercus_ (m^2^ ha^−1^)	StandDensity_Quercus_ (No. ha^−1^)
Kuandian	27.3±3.6de	12.2±2.4b	183.3±50.0b	4.0±1.6a	177.8±102.9a	6.9±1.8a	127.8±40.1a	3.4±1.5bc	116.7±44.1ab	0.0±0.0b	0.0±0.0b	4.7±0.9b	116.7±28.9b	1.0±1.0c	38.9±24.2ab
Dongsheng	31.5±2.9cd	20.6±5.0ab	233.3±53.6b	1.6±1.5ab	27.8±20.0b	5.6±3.6ab	55.6±14.7b	9.7±1.9ab	200.0±66.7a	0.0±0.0b	0.0±0.0b	5.5±2.8b	155.6±116.4b	17.0±9.7ab	105.6±65.5a
Liangshui	20.7±2.2e	21.8±4.6ab	483.3±171.1a	0.0±0.0b	5.6±5.6b	2.0±1.5bc	22.2±14.7bc	3.5±1.1bc	200.0±0.0a	0.0±0.0b	0.0±0.0b	0.3±0.0c	22.2±5.6b	11.7±5.2bc	111.1±36.4a
Shengshan	29.1±4.2de	21.8±0.9ab	316.7±58.3ab	0.2±0.2b	33.3±33.3b	1.3±1.3bc	16.7±16.7bc	0.9±0.3c	66.7±8.3bcd	0.4±0.4b	8.3±8.3b	1.8±1.0bc	16.7±8.3b	0.0±0.0c	0.0±0.0b
Changbai A	43.2±2.9ab	30.1±4.5a	188.9±33.8b	1.7±1.7ab	11.1±5.6b	0.0±0.0c	0.0±0.0c	11.7±4.0a	161.1±22.2ab	0.0±0.0b	0.0±0.0b	0.0±0.0c	0.0±0.0b	0.8±0.8c	5.6±5.6b
Changbai B	35.5±1.1bcd	18.1±2.2ab	166.7±9.6b	0.8±0.4b	27.8±14.7b	1.0±0.6bc	22.2±11.1bc	6.2±3.4abc	111.1±20.0abc	0.0±0.0b	0.0±0.0b	0.0±0.0c	0.0±0.0b	27.7±4.9a	111.1±5.6a
Changbai C	40.2±4.6bc	29.8±4.1a	205.6±14.7b	0.5±0.5b	5.6±5.6b	0.0±0.0c	0.0±0.0c	3.1±1.0bc	133.3±19.2abc	0.0±0.0b	0.0±0.0b	0.0±0.0c	0.0±0.0b	15.8±3.5ab	61.1±14.7ab
Changbai D	40.1±0.6bc	28.8±6.5a	211.1±49.4b	0.0±0.0b	0.0±0.0b	0.0±0.0c	0.0±0.0c	0.0±0.0c	5.6±5.6d	0.0±0.0b	0.0±0.0b	5.2±0.3b	566.7±83.9a	2.9±0.7c	50.0±9.6ab
Changbai E	50.2±4.6a	28.6±4.9a	133.3±19.2b	0.0±0.0b	0.0±0.0b	0.0±0.0c	0.0±0.0c	7.1±3.0abc	127.8±38.9abc	0.0±0.0bb	0.0±0.0b	11.7±1.6a	527.8±36.4a	0.0±0.0c	0.0±0.0b
Changbai F	33.4±1.5bcd	23.4±2.0ab	238.9±11.1b	0.0±0.0b	0.0±0.0b	0.4±0.4c	11.1±11.1bc	0.1±0.1c	44.4±14.7cd	15.6±3.5a	100.0±19.2a	14.6±2.4a	561.1±27.8a	0.0±0.0c	0.0±0.0b

Values represent mean ± standard error (n = 3). Different letters in each column indicate significant differences among sites (post-Duncan test, P<0.05).

### Responses of Composition and Structure in *Pinus Koraiensis* Forests to Climatic and Non-Climatic Factors

A few excluded independent variables showed significant partial correlations with the dependent variable (P<0.05, Table 4). The significant negative relationships of the BasalArea_Pinus_ with the climatic factors of PrecipWettestMonth and PrecipWettestQuarter, of the BasalArea_Betula_ and StandDensity_Betula_ with the climatic factors of PrecipDriestMonth and PrecipDriestQuarter, of the BasalArea_Betula_ with the climatic factor of TempColdestMonth, and of the StandDensity_Betula_ with the non-climatic factor of MeanDBH_Pinus_ were observed (P<0.05, Table 4).

**Table 4:** Matrix of two-tailed partial correlation coefficients between every dependent variable and the independent variables excluded from its model, controlling for the independent variable(s) included in its model.

	Temp Warmest Month	Temp Coldest Month	Temp Warmest Quarter	Temp Coldest Quarter	Precip Wettest Month	Precip Driest Month	Precip Wettest Quarter	Precip Driest Quarter	Mean DBH_Pinus_
BasalArea_Pinus_	0.17^NS^	−0.26^NS^	−0.08^NS^	−0.27^NS^	−0.39*	−0.36^NS^	−0.39*	−0.35^NS^	–
StandDensity_Pinus_	0.08^NS^	−0.18^NS^	0.02^NS^	−0.16^NS^	–	−0.11^NS^	−0.05^NS^	−0.10^NS^	–
BasalArea_Ulmus_	0.14^NS^	0.02^NS^	0.19^NS^	0.03^NS^	–	−0.10^NS^	−0.08^NS^	−0.05^NS^	−0.03^NS^
StandDensity_Ulmus_	0.09^NS^	−0.15^NS^	0.05^NS^	−0.16^NS^	–	−0.18^NS^	−0.23^NS^	−0.18^NS^	−0.18^NS^
BasalArea_Betula_	0.15^NS^	−0.38*	0.08^NS^	−0.36^NS^	–	−0.42*	−0.31^NS^	−0.40*	−0.30^NS^
StandDensity_Betula_	0.17^NS^	−0.36^NS^	0.09^NS^	−0.34^NS^	–	−0.41*	−0.33^NS^	−0.39*	−0.44*
BasalArea_Acer-Fraxinus_	0.10^NS^	−0.21^NS^	–	−0.19^NS^	−0.08^NS^	−0.19^NS^	−0.09^NS^	−0.17^NS^	–
StandDensity_Acer-Fraxinus_	0.16^NS^	−0.22^NS^	–	−0.20^NS^	−0.15^NS^	−0.24^NS^	−0.15^NS^	−0.21^NS^	0.11^NS^
BasalArea_Quercus_	–	0.30^NS^	0.14^NS^	0.29^NS^	−0.09^NS^	0.06^NS^	−0.05^NS^	0.08^NS^	−0.05^NS^
StandDensity_Quercus_	0.19^NS^	−0.15^NS^	–	−0.14^NS^	−0.24^NS^	−0.22^NS^	−0.23^NS^	−0.24^NS^	−0.33^NS^
BasalArea_Larix_	−0.19^NS^	0.22^NS^	–	0.22^NS^	0.11^NS^	0.18^NS^	0.15^NS^	0.22^NS^	−0.29^NS^
StandDensity_Larix_	−0.16^NS^	0.17^NS^	–	0.17^NS^	0.12^NS^	0.15^NS^	0.15^NS^	0.20^NS^	−0.35^NS^
BasalArea_Abies-Picea_	–	−0.28^NS^	−0.26^NS^	−0.26^NS^	−0.14^NS^	−0.22^NS^	−0.14^NS^	−0.21^NS^	−0.19^NS^
StandDensity_Abies-Picea_	–	−0.09^NS^	−0.28^NS^	−0.10^NS^	−0.33^NS^	−0.18^NS^	−0.30^NS^	−0.22^NS^	0.02^NS^

“–” indicates controlling for that variable. Significance level:^ NS^ (Non-significant) P>0.05.

*P<0.05.

**P<0.01 (n = 30).

Significant regression models were observed for all the 14 dependent variables (P<0.05, Table 5). MeanDBH_Pinus_ had positive relationships with BasalArea_Pinus_ and BasalArea_Acer-Fraxinus_, and had negative relationship with StandDensity_Pinus_ when the MeanDBH_Pinus_ was below 47.6 cm. PrecipWettestMonth suppressed StandDensity_Pinus_, while promoted BasalArea_Ulmus_, StandDensity_Ulmus_, BasalArea_Betula_ and StandDensity_Betula_. TempWarmestQuarter enhanced BasalArea_Acer-Fraxinus_, StandDensity_Acer- Fraxinus_ and StandDensity_Quercus_, but suppressed BasalArea_Larix_ and StandDensity_Larix_. TempWarmestMonth promoted BasalArea_Quercus_, but suppressed BasalArea_Abies-Picea_ and StandDensity_Abies-Picea_ (Table 5).

**Table 5:** Significant mathematical equations and regression coefficients (a, b, c, d) used to predict structure and composition of *Pinus koraiensis* mixed forests from linear and quadratic components of MeanDBH_Pinus_, PrecipWettestMonth, TempWarmestQuater and TempWarmestMonth.

Dependent Variable, Y	Model Form	Coefficients				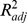
		a (S.E.)	b (S.E.)	c (S.E.)	d (S.E.)	
BasalArea_Pinus_	Y = a+b(MeanDBH_Pinus_)^2^	14.740 (2.719)***	0.007 (0.002)**	–	–	0.30**
StandDensity_Pinus_	Y = a+b(PrecipWettestMonth)^2^+c(MeanDBH_Pinus_)+d(MeanDBH_Pinus_)^2^	1232.331 (183.706)***	−0.003 (0.001)**	−42.803 (9.983)***	0.450 (0.136)**	0.61***
BasalArea_Ulmus_	Y = a+b(PrecipWettestMonth)	−3.786 (1.222)**	0.026(0.007)***	–	–	0.33***
StandDensity_Ulmus_	Y = a+b(PrecipWettestMonth)^2^	−60.350(20.458)**	0.003(0.001)***	–	–	0.45***
BasalArea_Betula_	Y = a+b(PrecipWettestMonth)	−6.445(2.200)**	0.046(0.012)***	–	–	0.32***
StandDensity_Betula_	Y = a+b(PrecipWettestMonth)	−129.583(23.427)***	0.874(0.129)***	–	–	0.61***
BasalArea_Acer-Fraxinus_	Y = a+b(TempWarmestQuater)+c(MeanDBH_Pinus_)^2^	−52.679(18.877)**	3.018(1.057)**	0.004(0.001)**	–	0.29**
StandDensity_Acer-Fraxinus_	Y = a+b(TempWarmestQuater)^ 2^	−313.895(142.855)*	1.430(0.473)**	–	–	0.22**
BasalArea_Quercus_	Y = a+b(TempWarmestMonth)	−161.720(57.358)**	7.033(2.380)**	–	–	0.21**
StandDensity_Quercus_	Y = a+b(TempWarmestQuater)	−664.410(213.063)**	41.112(12.278)**	–	–	0.26**
BasalArea_Larix_	Y = a+b(TempWarmestQuater)	63.876(17.998)**	−3.592(1.037)**	–	–	0.28**
StandDensity_Larix_	Y = a+b(TempWarmestQuater)	430.450 (110.937)***	−24.204 (6.393)***	–	–	0.32***
BasalArea_Abies-Picea_	Y = a+b(TempWarmestMonth)	141.160 (18.479)***	−5.679 (0.767)***	–	–	0.65***
StandDensity_Abies-Picea_	Y = a+b(TempWarmestMonth)	6330.509 (946.593)***	−254.657 (39.280)***	–	–	0.59***

Equations from stepwise regression analyses (n = 30). S.E., standard error; 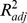, adjusted multiple coefficient of determination. Significance level:^ NS^ (Non-significant) P>0.05.

*P<0.05.

**P<0.01.

***P<0.001.

### Model Outputs

**Figure 2:**
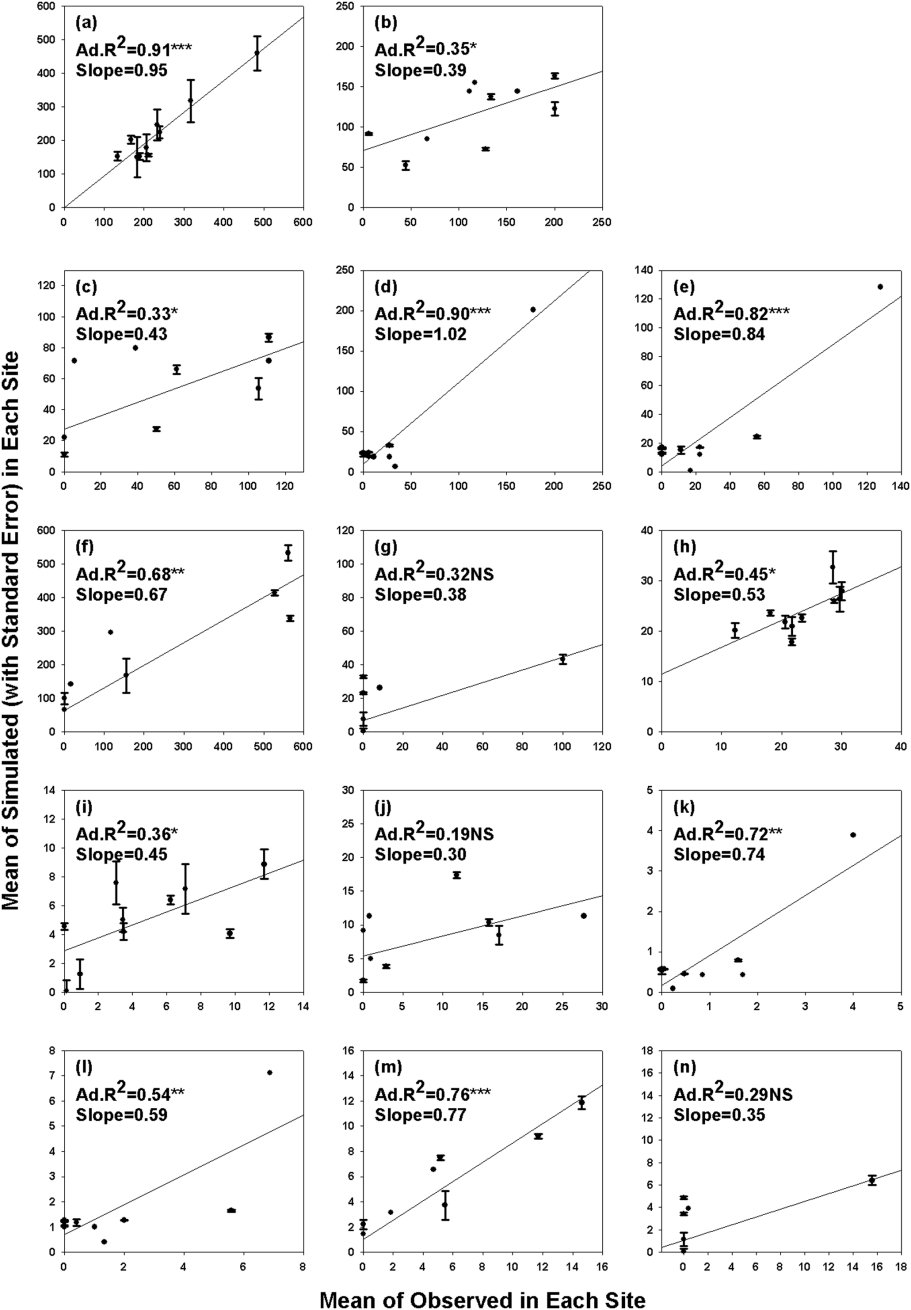
Linear regression relationships between the simulated (n = 3, mean with standard error) and observed (mean of each site) values of StandDensity_Pinus_ (No. ha^−1^; a), StandDensity_Acer-Fraxinus_ (No. ha^−1^; b), StandDensity_Quercus_ (No. ha^−1^; c), StandDensity_Ulmus_ (No.ha^−1^; d), StandDensity_Betula_ (No.ha^−1^; e), StandDensity_Abies-Picea_ (No. ha^−1^; f), StandDensity_Larix_ (No. ha^−1^; g), BasalArea_Pinus_ (m^2^ ha^−1^; h), BasalArea_Acer-Fraxinus_ (m^2^ ha^−1^; i), BasalArea_Quercus_ (m^2^ ha^−1^; j), BasalArea_Ulmus_ (m^2^ ha^−1^; k), BasalArea_Betula_ (m^2^ ha^−1^; l), BasalArea_Abies-Picea_ (m^2^ ha^−1^; m) and BasalArea_Larix_ (m^2^ ha^−1^; n) in *Pinus koraiensis* mixed forests. Significance level: ^NS^(Non-significant)P>0.05, *P<0.05, **P<0.01, ***P<0.001.

There were better concordances between observed and simulated values in stand densities than in basal areas. More significant linear regression relationships between observed and simulated values with higher adjusted R squares and closer-to-1 slopes were found in StandDensity_Pinus_ (Fig. 2a), StandDensity_Quercus_ (Fig. 2c), StandDensity_Ulmus_ (Fig. 2d) and StandDensity_Betula_ (Fig. 2e), compared with those in BasalArea_Pinus_ (Fig. 2h), BasalArea_Quercus_ (Fig. 2j), BasalArea_Ulmus_ (Fig. 2k) and BasalArea_Betula_ (Fig. 2l). However, better linear regression relationships between simulated and observed values were found in BasalArea_Acer-Fraxinus_ (Fig. 2i) and BasalArea_Abies-Picea_ (Fig. 2m), compared with those in StandDensity_Acer-Fraxinus_ (Fig. 2b) and StandDensity_Abies-Picea_ (Fig. 2f). The linear regression relationships between simulated and observed values in StandDensity_Larix_ (Fig. 2g) and BasalArea_Larix_ (Fig. 2n) were not significant.

**Figure 3:**
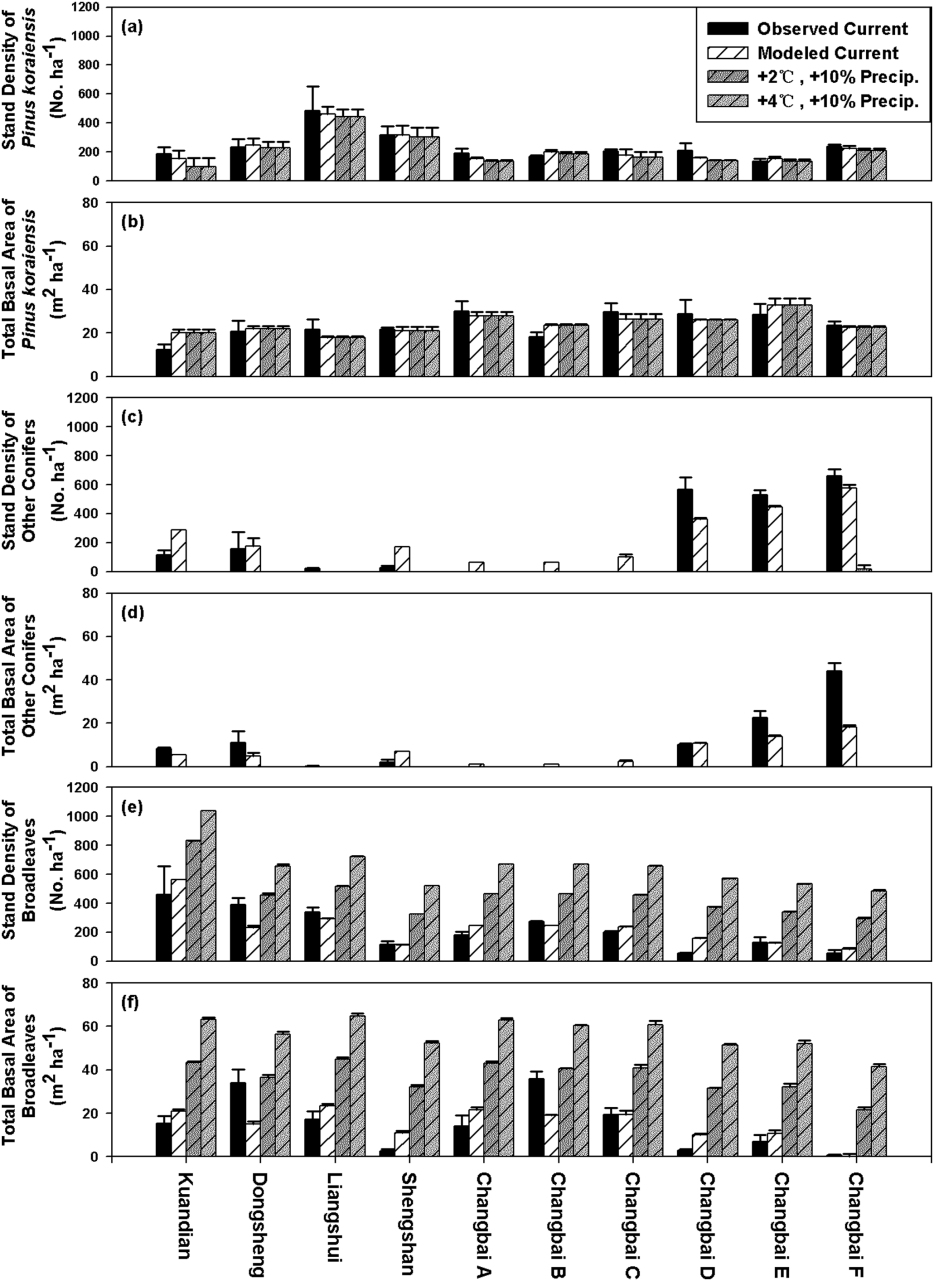
Stand density of *Pinus koraiensis* (a), total basal area of *Pinus koraiensis* (b), stand density of other conifers (c), total basal area of other conifers (d), stand density of broadleaves (e), total basal area of broadleaves (f) of *Pinus koraiensis* and other conifers (*Abies* and *Picea* and *Larix*) and broadleaf trees (broadleaves) in the ten sites (from left to right: Kuandian, Dongsheng, Liangshui, Shengshan and Changbai from A to F) observed under current condition and modeled under current climate and under +2°C and +10% precipitation scenario and under +4°C and +10% precipitation scenario (using the currently observed MeanDBH_Pinus_ values).

Stand densities and total basal areas of conifers were predicted to decrease while stand density and total basal area of broadleaved trees were predicted to increase under the +2°C and +10% precipitation and +4°C and +10% precipitation scenarios. Stand density of *Pinus koraiensis* was predicted to notably decrease in Kuandian and slightly decrease in other sites (Fig. 3a). Total basal area of *Pinus koraiensis* was predicted to keep constant (Fig. 3b). Stand density and total basal area of other conifers (*Abies* and *Picea* and *Larix*) were predicted to be zero (Fig. 3c and d). Broadleaved trees would increase dramatically (Fig. 3e and f). In all the ten sites, the densities and total basal areas of broadleaved trees would be higher than those of *Pinus koraiensis* under the +2°C and +10% precipitation and +4°C and +10% precipitation scenarios (Fig. 3).

Under each climate scenario, the stand density of *Pinus koraiensis* (StandDensity_Pinus_) firstly increased and then decreased with increasing latitude, while the stand density of broadleaved trees decreased with increasing latitude and altitude (Fig. 3).

## Discussion

*Betula* and *Ulmus* serve as early successional species (pioneers), while *Acer* and *Fraxinus* and *Pinus koraiensis* serve as late successional species during the succession of *Pinus koraiensis* mixed forest [1], [22]. In our results, MeanDBH_Pinus_ (which represents the stand age of a *Pinus koraiensis* forest) [16] was observed to have positive relationships with BasalArea_Pinus_ and BasalArea_Acer-Fraxinus_, suggesting that *Pinus koraiensis* and *Acer* and *Fraxinus* are late successional species and naturally increase in their total basal areas over time. However, negative relationship existed between MeanDBH_Pinus_ and StandDensity_Pinus_ when MeanDBH_Pinus_ was below 47.6 cm (Table 5), which indicated the self-thinning phenomenon of *Pinus* [23].

Heavier rain usually results in higher soil wetness [24]. Soil wetness greatly reduces soil cohesion and increases the possibility of tree uprooting [25]. Uprooting of trees results in exposed mineral soil (bare soil) [26], [27]. The exposed mineral soil favors the regeneration of pioneer trees such as *Betula* [28] and *Ulmus* [29]. In our results, PrecipWettestMonth was observed to have negative effect on StandDensity_Pinus_, but positive effects on BasalArea_Ulmus_, StandDensity_Ulmus_, BasalArea_Betula_ and StandDensity_Betula_ (Table 5). In addition, better concordances between simulated and observed values were found in StandDensity_Pinus_, StandDensity_Ulm_ and StandDensity_Betula_, compared with those in BasalArea_Pinus_, BasalArea_Ulm_ and BasalArea_Betula_ (Fig. 2). Our results indicate that higher monthly precipitations can cause higher soil wetnesses, more uprooting events and more exposed mineral soil, and therefore negatively modulate the StandDensity_Pinus_ and positively modulate the stand densities of *Betula* and *Ulmus*.

Climate warming has positive and negative effects on plants. Prolonged growing seasons and increased growing-season temperatures have been found to enhance the growth of some plants, especially the growth of many deciduous broadleaved trees [30], [31]. But during midday of summer, high temperature can inhibit the photosynthesis and growth of plants by provoking leaf-to-air vapor pressure deficit (VPD) [32], [33], [34]. Generally, broadleaf trees are less sensitive than conifers trees [35] while *Pinus* trees are less sensitive than other conifer trees (*Picea* and *Larix*) [36], [37] to the leaf-to-air VPDs. In our result, no effects of temperature on *Pinus koraiensis* but the negative effects of summer temperatures on other conifers (*Abies* and/or *Picea* and *Larix*) and the positive effects of summer temperatures on some broadleaved trees (*Acer* and/or *Fraxinus* and *Quercus*) were observed (Table 5). Our results indicate that (1) hotter summers have hotter middays and thus more severe leaf-to-air VPDs, which are more beneficial to the growth of broadleaf trees compared with conifer trees, and more beneficial to the growth of *Pinus koraiensis* trees compared with other conifer trees (*Abies* and/or *Picea* and *Larix*); (2) increased summer (growing-season) temperatures may enhance the growths of many broadleaved trees (*Acer* and/or *Fraxinus* and *Quercus*).

A wide-ranged pine species is usually composed of many genetically-different local populations. Each local population has evolved to adapt to its local climate, and has its unique response curve to climate change [11], [38]. In our results, no effect of temperature on the density and total basal area of *Pinus koraiensis* were observed (Table 5), and the reason may possibly is due to the local adaptations of many genetically-different *Pinus koraiensis* populations. We suggest that the *Pinus koraiensis* in the region may not necessarily maintain itself under the +2°C and +10% precipitation and +4°C and +10% precipitation scenarios if proper migrations of local populations do not occur in time.

Studies using a forest gap model alone usually predicted the extinction of *Pinus koraiensis* within 150 years in current *Pinus koraiensis* mixed forest under climate warming scenarios [7], [39], while other study linking a forest gap model with a landscape model predicted that the *Pinus koraiensis* could persist for at least 300 years under the warming climate [7]. Our result agreed with the latter by predicting that *Pinus koraiensis* would persist, while the broadleaf trees would overtake *Pinus koraiensis* to become dominant species under either of the two warming scenarios (Fig. 3).

## Conclusions

These results show that some mathematical models were successfully built to quantify the responses of the composition and structure of *Pinus koraiensis* mixed forests to the climatic factors. TempWarmestMonth, TempWarmestQuarter and PrecipWettestMonth strongly affected the stand densities and total basal areas in *Pinus koraiensis* mixed forest and therefore they are suggested to be key climatic factors which shape the composition and structure of *Pinus koraiensis* mixed forest. The composition and structure of *Pinus koraiensis* mixed forests were significantly affected, and although *Pinus koraiensis* would persist, the current forests dominated by *Pinus koraiensis* in the region would all shift and become broadleaf-dominated forests due to the dramatic increase of broadleaf trees under the future global warming and increased precipitations.
